# Supplementary material for: Sociodemographic Predictors of Changes in Physical Activity, Screen Time, and Sleep among Toddlers and Preschoolers in Chile during the COVID-19 Pandemic
Source: Int J Environ Res Public Health. 2020 Dec 29;18(1):176. doi: 10.3390/ijerph18010176 (PMC7796176; doi:10.3390/ijerph18010176)
Supplement: Supplementary file 1 [file ijerph-18-00176-s001.zip › Supplementary table 1 - descriptive.docx]

Supplementary Table 1. Description of movement behaviors before and during the early stages of the COVID-19 pandemic in toddlers and pre-schoolers in Chile according to sociodemographic factors.

|  | Physical activity  (mean [SD], h/day) | | | Screen time  (mean [SD], h/day) | | | Sleep duration  (mean [SD], h/day) | | | Sleep quality  (mean [SD], score 1 to 7) | | |
| --- | --- | --- | --- | --- | --- | --- | --- | --- | --- | --- | --- | --- |
| Characteristic | Before COVID-19 | During COVID-19 | p | Before COVID-19 | During COVID-19 | p | Before COVID-19 | During COVID-19 | p | Before COVID-19 | During COVID-19 | p |
| Family income |  |  |  |  |  |  |  |  |  |  |  |  |
| <530 USD | 3.70^a^  (2.10) | 2.98^b^  (2.26) | **<0.001** | 1.74^a^  (1.26) | 3.08  (2.08) | **<0.001** | 10.92  (2.07) | 11.00  (2.11) | 0.160 | 5.62  (1.69) | 4.74^b^  (1.90) | **<0.001** |
| ≥530-<1830 USD | 3.58  (1.96) | 2.80  (2.12) | **<0.001** | 1.67  (1.15) | 3.09  (1.89) | **<0.001** | 10.93  (1.68) | 11.03  (1.81) | **0.015** | 5.69  (1.48) | 4.94  (1.72) | **<0.001** |
| ≥1830 USD | 3.34  (1.75) | 2.60  (1.98) | **<0.001** | 1.48  (0.92) | 2.90  (1.73) | **<0.001** | 10.87  (1.56) | 10.97  (1.50) | 0.064 | 5.73  (1.45) | 5.23  (1.62) | **<0.001** |
| Main caregiver’s level of education |  |  |  |  |  |  |  |  |  |  |  |  |
| Incomplete high school | 3.58^a^  (2.20) | 3.26^b^  (2.57) | 0.124 | 1.96^a^  (1.46) | 2.99  (2.20) | **<0.001** | 10.81  (2.68) | 10.99  (2.79) | 0.431 | 5.21^a^  (1.95) | 4.39^b^  (2.20) | **<0.001** |
| Complete high school | 3.72  (2.11) | 3.07  (2.30) | **<0.001** | 1.73  (1.24) | 3.04  (1.93) | **<0.001** | 10.93  (1.93) | 11.10  (1.96) | **0.005** | 5.63  (1.64) | 4.81  (1.86) | **<0.001** |
| Technical degree | 3.52  (2.01) | 2.74  (2.11) | **<0.001** | 1.74  (1.25) | 3.23  (2.06) | **<0.001** | 10.93  (1.74) | 10.94  (1.93) | 0.952 | 5.73  (1.55) | 4.87  (1.78) | **<0.001** |
| University degree | 3.51  (1.86) | 2.67  (2.01) | **<0.001** | 1.57  (1.04) | 3.01  (1.87) | **<0.001** | 10.91  (1.66) | 10.97  (1.71) | 0.067 | 5.72  (1.45) | 5.05  (1.67) | **<0.001** |
| Children enrolled in ECEC |  |  |  |  |  |  |  |  |  |  |  |  |
| 1- to 2-year old |  |  |  |  |  |  |  |  |  |  |  |  |
| Yes | 4.10^a^  (2.21) | 3.31  (2.37) | **<0.001** | 1.37^a^  (1.03) | 2.66^b^  (1.77) | **<0.001** | 11.58^a^  (1.75) | 11.64  (1.91) | 0.372 | 5.52^a^  (1.50) | 4.75  (1.75) | **<0.001** |
| No | 3.71  (2.11) | 3.46  (2.36) | **<0.001** | 1.57  (1.15) | 2.25  (1.63) | **<0.001** | 11.85  (2.01) | 11.64  (2.10) | **0.003** | 5.27  (1.63) | 4.67  (1.81) | **<0.001** |
| 3- to 5-year old |  |  |  |  |  |  |  |  |  |  |  |  |
| Yes | 3.34  (1.79) | 2.45  (1.91) | **<0.001** | 1.77  (1.16) | 3.46^b^  (1.97) | **<0.001** | 10.38^a^  (1.55) | 10.58  (1.65) | **<0.001** | 5.87^a^  (1.48) | 5.07  (1.74) | **<0.001** |
| No | 3.50  (1.89) | 2.73  (1.95) | **<0.001** | 1.98  (1.36) | 2.89  (1.74) | **<0.001** | 10.82  (1.54) | 10.85  (1.58) | 0.839 | 5.56  (1.77) | 4.98  (1.94) | **<0.001** |
| Dwelling type |  |  |  |  |  |  |  |  |  |  |  |  |
| House | 3.57  (1.95) | 2.91^b^  (2.12) | **<0.001** | 1.66  (1.17) | 3.01^b^  (1.92) | **<0.001** | 10.91  (1.80) | 11.02  (1.85) | **<0.001** | 5.67  (1.55) | 4.95  (1.76) | **<0.001** |
| Apartment | 3.52  (1.97) | 2.22  (2.07) | **<0.001** | 1.67  (1.10) | 3.38  (1.91) | **<0.001** | 10.96  (1.72) | 10.91  (1.86) | 0.494 | 5.73  (1.48) | 4.83  (1.80) | **<0.001** |
| Other | 3.92  (2.43) | 3.40  (2.49) | **0.007** | 1.54  (1.02) | 2.51  (1.79) | **<0.001** | 11.00  (1.93) | 11.13  (2.15) | 0.427 | 5.55  (1.69) | 4.83  (1.92) | **<0.001** |
| Home size |  |  |  |  |  |  |  |  |  |  |  |  |
| <50 m^2^ | 3.67  (2.11) | 2.94^b^  (2.32) | **<0.001** | 1.77^a^  (1.32) | 3.15^a^  (2.12) | **<0.001** | 10.99  (1.98) | 11.05  (2.03) | 0.291 | 5.66  (1.57) | 4.73^b^  (1.86) | **<0.001** |
| 50 - <100 m^2^ | 3.58  (1.97) | 2.70  (2.10) | **<0.001** | 1.61  (1.09) | 3.08  (1.90) | **<0.001** | 10.87  (1.78) | 10.96  (1.86) | **0.024** | 5.68  (1.55) | 4.95  (1.75) | **<0.001** |
| ≥100 m^2^ | 3.49  (1.82) | 2.97  (2.04) | **<0.001** | 1.63  (1.08) | 2.88  (1.74) | **<0.001** | 10.94  (1.62) | 11.06  (1.67) | **0.024** | 5.70  (1.49) | 5.10  (1.70) | **<0.001** |
| Number of people per home |  |  |  |  |  |  |  |  |  |  |  |  |
| ≤3 | 3.57  (1.92) | 2.77^b^  (2.10) | **<0.001** | 1.60^a^  (1.09) | 3.05  (1.89) | **<0.001** | 11.07^a^  (1.77) | 11.16^b^  (1.85) | 0.066 | 5.66  (1.49) | 4.95  (1.74) | **<0.001** |
| 4 | 3.49  (1.92) | 2.74  (2.13) | **<0.001** | 1.65  (1.20) | 3.04  (1.89) | **<0.001** | 10.83  (1.78) | 10.86  (1.83) | 0.553 | 5.75  (1.51) | 5.00  (1.70) | **<0.001** |
| ≥5 | 3.67  (2.09) | 2.98  (2.21) | **<0.001** | 1.73  (1.17) | 3.05  (1.99) | **<0.001** | 10.82  (1.83) | 10.99  (1.90) | **0.002** | 5.62  (1.64) | 4.83  (1.87) | **<0.001** |
| Children per home |  |  |  |  |  |  |  |  |  |  |  |  |
| 1 child | 3.70^a^  (1.99) | 2.86  (2.12) | **<0.001** | 1.61^a^  (1.08) | 2.97^b^  (1.85) | **<0.001** | 11.06^a^  (1.83) | 11.11^b^  (1.84) | 0.272 | 5.64  (1.50) | 4.91  (1.75) | **<0.001** |
| 2 children | 3.45  (1.95) | 2.78  (2.17) | **<0.001** | 1.66  (1.18) | 3.09  (1.90) | **<0.001** | 10.82  (1.75) | 10.91  (1.84) | 0.057 | 5.75  (1.53) | 4.96  (1.74) | **<0.001** |
| 3 or more | 3.51  (1.94) | 2.83  (2.18) | **<0.001** | 1.81  (1.29) | 3.22  (2.20) | **<0.001** | 10.67  (1.76) | 10.93  (1.98) | **0.001** | 5.60  (1.71) | 4.89  (1.91) | **<0.001** |
| Squared meters per person at home |  |  |  |  |  |  |  |  |  |  |  |  |
| <11.7 m^2^ per person | 3.67  (2.14) | 2.93  (3.30) | **<0.001** | 1.76^a^  (1.33) | 3.09  (2.12) | **<0.001** | 10.87  (1.96) | 11.00  (2.02) | **0.033** | 5.63  (1.65) | 4.72^b^  (1.89) | **<0.001** |
| ≥11.7 - <18.3 m^2^ per person | 3.62  (2.06) | 2.79  (2.19) | **<0.001** | 1.68  (1.15) | 3.12  (1.93) | **<0.001** | 10.91  (1.78) | 10.92  (1.91) | 0.948 | 5.64  (1.56) | 4.85  (1.75) | **<0.001** |
| ≥18.3 - <25 m^2^ per person | 3.48  (1.86) | 2.70  (2.04) | **<0.001** | 1.64  (1.04) | 3.10  (1.90) | **<0.001** | 10.89  (1.80) | 10.98  (1.85) | 0.124 | 5.71  (1.50) | 4.98  (1.72) | **<0.001** |
| ≥25 m^2^ per person | 3.54  (1.83) | 2.86  (2.05) | **<0.001** | 1.56  (1.06) | 2.91  (1.74) | **<0.001** | 10.98  (1.65) | 11.10  (1.67) | **0.011** | 5.72  (1.47) | 5.13  (1.69) | **<0.001** |
| Available space to play |  |  |  |  |  |  |  |  |  |  |  |  |
| Yes | 3.57  (1.95) | 2.88^b^  (2.14) | **<0.001** | 1.65  (1.15) | 2.99^b^  (1.88) | **<0.001** | 10.95^a^  (1.79) | 11.02  (1.85) | **0.011** | 5.70^a^  (1.52) | 4.99^b^  (1.75) | **<0.001** |
| No | 3.72  (2.21) | 2.12  (2.12) | **<0.001** | 1.80  (1.18) | 3.77  (2.29) | **<0.001** | 10.50  (1.83) | 10.80  (2.00) | **0.015** | 5.42  (1.82) | 4.16  (1.85) | **<0.001** |
| Living area |  |  |  |  |  |  |  |  |  |  |  |  |
| Urban | 3.57  (1.98) | 2.71^b^  (2.10) | **<0.001** | 1.67  (1.16) | 3.11^b^  (1.94) | **<0.001** | 10.91  (1.78) | 10.98^b^  (1.84) | **0.014** | 5.69  (1.54) | 4.91  (1.78) | **<0.001** |
| Rural | 3.63  (1.94) | 3.68  (2.28) | 0.576 | 1.58  (1.13) | 2.58  (1.71) | **<0.001** | 10.99  (1.90) | 11.20  (2.04) | **0.005** | 5.60  (1.59) | 5.04  (1.72) | **<0.001** |
| Lockdown |  |  |  |  |  |  |  |  |  |  |  |  |
| Yes | 3.56  (1.96) | 2.77^b^  (2.12) | **<0.001** | 1.63^a^  (1.13) | 3.05  (1.88) | **<0.001** | 10.95  (1.80) | 11.00  (1.85) | 0.116 | 5.69  (1.51) | 4.92  (1.78) | **<0.001** |
| No | 3.64  (2.02) | 2.98  (2.23) | **<0.001** | 1.75  (1.22) | 3.06  (2.04) | **<0.001** | 10.81  (1.78) | 11.03  (1.89) | **<0.001** | 5.63  (1.64) | 4.96  (1.76) | **<0.001** |

Abbreviations: COVID-19, coronavirus disease 2019; ECEC, early childhood care and education centres; USD, United States dollar.

^a^: p-value <0.05 when comparing each behaviour between categories before COVID-19

^b^: p-value <0.05 when comparing each behaviour between categories during COVID-19
