# Supplementary material for: Sociodemographic Predictors of Changes in Physical Activity, Screen Time, and Sleep among Toddlers and Preschoolers in Chile during the COVID-19 Pandemic
Source: Int J Environ Res Public Health. 2020 Dec 29;18(1):176. doi: 10.3390/ijerph18010176 (PMC7796176; doi:10.3390/ijerph18010176)
Supplement: Supplementary file 1 [file ijerph-18-00176-s001.zip › Supplementary file 1 - Questions about Movement Behaviours.docx]

**Supplementary file 1. Movement behaviours related questions included in the study**

This section of the questionnaire will ask about movement behaviours BEFORE the closure of educational centres (before March 16th) and then, about movement behaviours AFTER the closure of them (Monday, March 16th to date). Please, record the hours and minutes as accurately as possible.

1. PHYSICAL ACTIVITY
   1. On a typical day, BEFORE the educational centres closed (before March 16th), how much time in total do you estimate the child spent doing physical activities, considering all they do in a day? Include the time you estimate both in the educational centre (if the child attends one) and at home. (For example: if the child spent 40 minutes playing with a ball in the morning and then, in the afternoon, he was playing jumping for 30 minutes, the total for that day would be 1 hour and 10 minutes)
   2. And now thinking AFTER the educational centres closed (Monday, March 16th to date), how much time in total do you estimate that the child spends doing physical activities, considering all they do in a day? (For example: if the child spent 40 minutes playing with a ball in the morning and then, in the afternoon, he was playing jumping for 30 minutes, the total for that day would be 1 hour and 10 minutes)
2. SCREEN ELECTRONIC DEVICES
   1. On a typical day, BEFORE educational centres closed (before March 16th), how much time in total do you estimate the child spent using a touch screen electronic device such as a smartphone, tablet, playing video games, watching TV, movies or videos on the internet while sitting or lying down? (Think of the child's main activity. For example, if the TV was on, but the child was concentrating on playing without looking at it, do not count it)
   2. And now thinking AFTER the educational centres closed (Monday, March 16th to date), how much time do you estimate the child spends using an electronic touch screen device such as a smartphone, a tablet, a video game, watching TV, movies or videos on the internet while sitting or lying down? (Think of the child's main activity. For example, if the TV was on, but the child was concentrating on playing without looking at it, do not count it)
3. SLEEP
   1. On a typical day, BEFORE educational centres closed (before March 16th), how much time do you estimate the child slept per day? Include naps and the night.
   2. And now thinking AFTER the educational centres closed (Monday, March 16th to date), how much time do you estimate the child sleep per day? Include naps and the night.
4. SLEEP QUALITY
   1. On a typical day, BEFORE educational centres closed (before March 16th), how would you assess the sleep quality of the child?

"1" would indicate difficulty falling asleep, waking up numerous times during the night for long periods and restless (cough and turns, moves and bedclothes off), while "7" would indicate falling asleep without problems and in a few minutes, sleeping well at night, good breathing and deep sleeping.

- 1. And now thinking AFTER the educational centres closed (Monday, March 16th to date), how would you assess the sleep quality of the child?

"1" would indicate difficulty falling asleep, waking up numerous times during the night for long periods and restless (cough and turns, moves and bedclothes off), while "7" would indicate falling asleep without problems and in a few minutes, sleeping well at night, good breathing and deep sleeping.

**Suplemento 1. Preguntas sobre conductas físicas incluidas en el estudio.**

Esta sección del cuestionario preguntará de forma consecutiva sobre conductas realizadas ANTES del cierre de establecimientos educacionales (antes del 16 de marzo), y luego sobre conductas realizadas DESPUÉS del cierre de establecimientos (lunes 16 de marzo hasta la fecha). Por favor, registre con la máxima exactitud que pueda las horas y los minutos.

1. ACTIVIDAD FÍSICA
   1. En un día típico, ANTES de que cerraran los establecimientos educacionales/de cuidados (antes del 16 de marzo), ¿cuánto tiempo en total estima usted que el niño/a pasó realizando actividades físicas, considerando todas las que hace en un día? Incluya el tiempo que estima tanto en el establecimiento educacional/de cuidados (en caso de que asista a uno), como en el hogar.

(Por ejemplo: si el niño/a pasó jugando con una pelota en la mañana 40 minutos y luego en la tarde estuvo jugando a saltar por 30 minutos, el total de ese día seria 1 hora y 10 minutos)

- 1. Y ahora pensando DESPUÉS de que cerraron los establecimientos educacionales/de cuidados (lunes 16 de marzo hasta la fecha), ¿cuánto tiempo en total estima usted que el niño/a pasa realizando actividades físicas, considerando todas las que hacen un día? (Por ejemplo: si el niño/a pasó jugando con una pelota en la mañana 40 minutos y luego en la tarde estuvo jugando a saltar por 30 minutos, el total de ese día seria 1 hora y 10 minutos)

1. DISPOSITIVOS ELECTRÓNICOS CON PANTALLA
   1. En un día típico, ANTES de que cerraran los establecimientos educacionales (antes del 16 de marzo), ¿cuánto tiempo estima usted que pasó el niño/a utilizando un dispositivo electrónico de pantalla táctil como un teléfono inteligente, una tablet, jugando videojuegos, viendo televisión o películas, videos en internet mientras estuvieron sentados o recostados?

(Piense en la actividad principal del niño/a. Por ejemplo, si la televisión estaba encendida, pero el niño/a estaba concentrado jugando sin mirarla, no lo contabilice)

- 1. Y ahora pensando DESPUÉS de que cerraron los establecimientos educacionales (lunes 16 de marzo hasta la fecha), ¿cuánto tiempo estima usted que pasa el niño/a utilizando un dispositivo electrónico de pantalla táctil como un teléfono inteligente, una tablet, un videojuego, viendo televisión o películas, videos en internet mientras estuvieron sentados o recostados?

(Piense en la actividad principal del niño/a. Por ejemplo, si la televisión estaba encendida, pero el niño/a estaba concentrado jugando, no lo contabilice)

1. SUEÑO
   1. En un día típico, ANTES de que cerraran los establecimientos educacionales (antes del 16 de marzo), ¿cuánto tiempo estima que durmió el niño/a por día? Incluya siestas y la noche.
   2. Y ahora pensando DESPUÉS de que cerraron los establecimientos educacionales (lunes 16 de marzo hasta la fecha), ¿cuánto tiempo estima que duerme el niño/a por noche? Incluya siestas y la noche.
2. CALIDAD DE SUEÑO
   1. En un día típico, ANTES de que cerraran los establecimientos educacionales (antes del 16 de marzo), ¿cómo evaluaría la calidad del sueño del niño(a)?

"1" indicaría dificultad para dormirse, que se despierta numerosas veces durante la noche por periodos largos y está muy inquieto (tose y se gira, mueve y se destapa), mientras "7" indicaría que se duerme sin problemas y en pocos minutos, que duerme bien durante la noche, que tiene un buen respirar y sueño profundo.

- 1. Y ahora pensando DESPUÉS de que cerraron los establecimientos educacionales (lunes 16 de marzo hasta la fecha), ¿cómo evaluaría la calidad del sueño del niño(a) durante la semana actual?

"1" indicaría dificultad para dormirse, que se despierta numerosas veces durante la noche por periodos largos y está muy inquieto (tose y se gira, mueve y se destapa), mientras "7" indicaría que se duerme sin problemas y en pocos minutos, que duerme bien durante la noche, que tiene un buen respirar y sueño profundo.
